# Improved Classification of Lung Cancer Tumors Based on Structural and Physicochemical Properties of Proteins Using Data Mining Models

**DOI:** 10.1371/journal.pone.0058772

**Published:** 2013-03-07

**Authors:** R. Geetha Ramani, Shomona Gracia Jacob

**Affiliations:** 1 Department of Information Science and Technology, College of Engineering, Guindy, Anna University, Chennai, Tamilnadu, India; 2 Faculty of Information and Communication Engineering, Anna University, Chennai, Tamilnadu, India; University of South Florida College of Medicine, United States of America

## Abstract

Detecting divergence between oncogenic tumors plays a pivotal role in cancer diagnosis and therapy. This research work was focused on designing a computational strategy to predict the class of lung cancer tumors from the structural and physicochemical properties (1497 attributes) of protein sequences obtained from genes defined by microarray analysis. The proposed methodology involved the use of hybrid feature selection techniques (gain ratio and correlation based subset evaluators with Incremental Feature Selection) followed by Bayesian Network prediction to discriminate lung cancer tumors as Small Cell Lung Cancer (SCLC), Non-Small Cell Lung Cancer (NSCLC) and the COMMON classes. Moreover, this methodology eliminated the need for extensive data cleansing strategies on the protein properties and revealed the optimal and minimal set of features that contributed to lung cancer tumor classification with an improved accuracy compared to previous work. We also attempted to predict via supervised clustering the possible clusters in the lung tumor data. Our results revealed that supervised clustering algorithms exhibited poor performance in differentiating the lung tumor classes. Hybrid feature selection identified the distribution of solvent accessibility, polarizability and hydrophobicity as the highest ranked features with Incremental feature selection and Bayesian Network prediction generating the optimal Jack-knife cross validation accuracy of 87.6%. Precise categorization of oncogenic genes causing SCLC and NSCLC based on the structural and physicochemical properties of their protein sequences is expected to unravel the functionality of proteins that are essential in maintaining the genomic integrity of a cell and also act as an informative source for drug design, targeting essential protein properties and their composition that are found to exist in lung cancer tumors.

## Introduction

Oncogenic tumors are the leading cause of death around the world with Lung Cancer bearing the major toll of malignant fatalities [1]–[3]. Smoking and use of tobacco along with diverse environmental carcinogens increased human susceptibility to this deadly ailment [4]–[5]. Gene Polymorphisms concerned with detoxification of carcinogens have been associated with formation of lung tumors. Lung tumors have been broadly categorized as Non-Small Cell Lung Cancer (NSCLC) affecting nearly two-thirds of patients with a low-survival rate and Small Cell Lung Cancer (SCLC), both of which respond to different forms of therapy [6]–[10]. This drives the need to precisely identify pathological differences between these two types of tumors.

Gene expression patterns from microarray analysis enabled the sub-categorization of lung cancer types that related to the degree of tumor demarcation, nature of therapy and victim survival rate [11]–[14]. It was an established fact that Lung carcinogenesis was a process that involved gradual phenotypic changes that occurred as a result of onco-gene activation and deactivation of tumor suppressor genes [8]. Reports thus far in literature have failed to identify any reliable biomarkers for this condition since wet-lab experiments often consumed more time, expertise and capital with unsure returns [1] [4]–[6]. Microarray technology has been utilized in the recent past to detect appropriate biomarkers but present methodologies were more susceptible to overlook potential facts contained in patient tissue samples [14]. Hence determination of potential and informative markers (diagnostic and prognostic) from both the biological and molecular perspective is highly essential to study and evaluate the genetic and molecular distinctiveness that characterized tumors and Tumor Node metastasis (TNM) staging in lung carcinogenesis to make possible effective diagnosis, and corroborate therapeutic strategies.

In recent research undertakings, several classifiers and data mining models have been used that targeted the appropriate categorization of lung cancer tumors. Forty-one samples characterized by 26 attributes computed from the mass-to-charge ratio (m/z) and peak heights of proteins identified by mass spectroscopy of blood serum samples from lung cancer affected and non-affected patients was utilized to train a classification and regression tree (CART) model [13]. Molecular classification of NSCLC based on a percentage train-test approach was used to evaluate the reliability of cDNA microarray-based classifications of resected human non-small cell lung cancers (NSCLCs) [14]. In further research Linear Discriminant Analysis and Artificial Neural Network classification of individual lung cancer cell lines (SCLC and NSCLC) was performed based on DNA methylation markers [13]. The results reported that Artificial Neural Network analysis of DNA methylation data was a potential technique to develop automated methods for lung cancer classification. In another study Support Vector Machine [14] was used in lung cancer gene expression database analysis and the results proposed that incorporated prior knowledge into cancer classification based on gene expression data was essential to improve classification accuracy. Automatic classification of lung TNM cancer stages from free-text pathology reports using symbolic rule- based classification was attempted [15]. The methodology was assessed based on accuracy parameters and confusion matrices against a database of multidisciplinary team staging by decisions and a machine learning-based text classification system using support vector machines.

The current investigation was focussed on a very recent article by Hosseinzadeh et.al [1] that aimed to classify lung cancer tumors based on structural and physiochemical properties of proteins using Bioinformatics models. We chose this paper for three main reasons. (i) The work is the most recent and the data is publicly available. (ii) The research involved plenty of data cleaning and pre-processing strategies which could be avoided. (iii) Their work involved few assumptions on the obtained data which are not adopted in this work. Moreover the method proposed in this paper was able to generate higher classification accuracy in differentiating between lung cancer tumors based on protein properties while retaining the original data and eliminating assumptions. Precisely this paper makes the following contributions: (a) Design of a new methodology with hybrid feature selection techniques to identify the optimal protein features that distinguished between lung cancer tumors with higher accuracy. (b) Eliminated the need for data cleaning and assumptions on attribute significance. (c) Contributing features identified are believed to influence drug design that could target the protein property leading to lung cancer tumors.

## Materials and Methods

### Dataset

The Gene Set Enrichment Analysis database (GSEA db) [16] was utilized to obtain the gene sets that contributed to the development of NSCLC and SCLC. It was obtained from the Kyoto Encyclopaedia of Genes and Genomes (KEGG) [17] gene sets. A total of 84 genes [17] were present in the SCLC gene set while 54 genes [17] were found contributing to NSCLC. In order to precisely discriminate between the two classes of tumors, the genes commonly occurring in both tumors were placed in a different class called COMMON. The strength of the gene set for SCLC was 59, NSCLC included 29 while the COMMON gene set summed up to 25. Proteins for each group of genes were obtained from the Gene Card database [18] and the corresponding protein sequences extracted from UniProt Knowledgebase database [19]. These sequences were saved as text file and loaded onto PROFEAT web server [20]–[21] to compute the structural and physicochemical properties associated with the protein. A total of one thousand four hundred and ninety seven attributes were computed and represented as Fi.j.k.l where ‘l’ represented the descriptor value and ‘k’ denoted the descriptor while ‘j’ indicated the feature and ‘í’ signified the feature group [20]–[21]. The features and their annotations have been provided as File S1. The complete data set comprising of 1497 features and 113 tumor samples [17] were loaded in to WEKA 3.7.7 machine learning software [22] and the tumor type was set to be the target class. The complete pre-processed dataset is provided as File S2. The variation in sample size as compared to previous work is attributed to possible updations in the database. The methodology proposed in this research work is described in the following section.

### Proposed Computational Methodology

**Figure 1:**
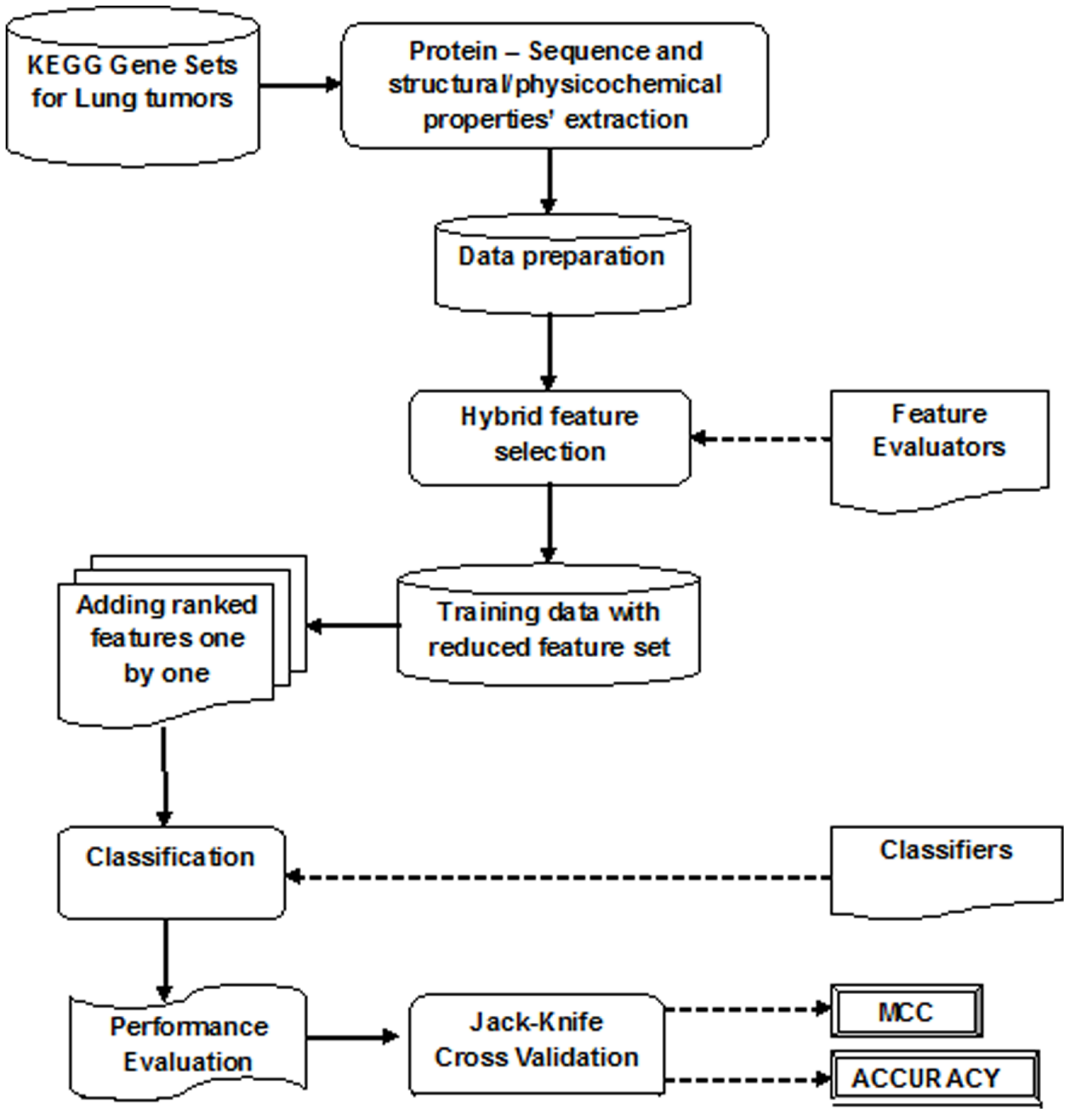
Proposed computational methodology for lung tumor classification from protein sequence properties.

The proposed methodology comprised of two phases: The training phase and the prediction phase. The training phase incorporated the data preparation, feature selection and classification process while the prediction phase involved evaluation of the classifier model using Jack-knife cross-validation test based on the performance parameters [23]–[24]: Matthews Correlation Co-efficient (MCC) and Accuracy. The diagrammatic representation of the proposed methodology is given in Figure 1. The data preparation phase incorporated categorization of the input gene sets as SCLC, NSCLC and the COMMON classes. This was followed by Hybrid feature selection with Incremental Feature Selection. The classification models were then built and compared to identify the best performing computational prediction technique on lung tumor classification using protein structural and physicochemical properties.

#### Hybrid Feature Selection

Feature ranking presented significant features in the order of their contribution to categorizing the samples under the different target classes [25]–[28]. Since most feature selection algorithms focused on ranking the attributes according to their significance value, the liability of choosing the limiting constraint rested with the user [29]–[31]. Hence in order to automate the process of finding the minimal yet optimal set of features, the ranking feature selection algorithms were followed by Correlation Subset Evaluators [32] that included features highly correlated to the class and least correlated to each other. Since both the ranking and subset evaluators were utilized to obtain the optimal feature set, this was termed the Hybrid Feature Selection strategy. The description of the methods used in this research is detailed below.

#### Gain Ratio Criterion

Gain ratio criterion [33]–[34], revealed the association between an attribute and the class value, being primarily computed from the Information Gain using the Information Entropy (InfoE) values [35]. After having obtained the value of the Entropy H(S_R_), and assuming ‘F’ to be the set of all features, and S_R_ to be the set of all records, Value(r,f) is taken to be the value of a specific instance ‘r <$>\raster="rg1"<$> S’ for the feature ‘f <$>\raster="rg1"<$> F’. Information Gain for the attribute was computed using Equation (1) as follows [35]:
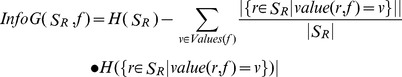
(1)


In order to compute the Intrinsic Value for a test, the following formula was adopted:
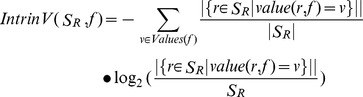
(2)


The Information Gain Ratio [33]–[35] was calculated as the ratio between the Information Gain and the Intrinsic value, according to Equation (3)
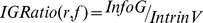
(3)


The attributes were thus ranked according to their rank in the descending order of the Gain Ratio score and were used for the CFS Subset Evaluator method described below.

#### Correlation Feature Selection (CFS) Subset Evaluator

The CFS hypothesis [36] suggested that the most predictive features needed to be highly correlated to the target class and least relevant to other predictor attributes. The following equation [36]–[37] recorded the value of a feature subset S that consisted of ‘k’ features
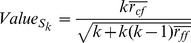
(4)where, 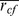 was the average value of all feature-classification correlations, and 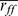 was the average value of all feature-feature correlations. The CFS criterion [36] was defined as follows:



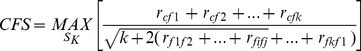
(5)Where 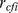
and 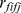
variables were referred to as correlations. The attributes that portrayed a high correlation to the target class and least relevance to each other were chosen as the best subset of attributes.

The attributes filtered by the CFS Subset Evaluator method were added in an incremental manner to identify the optimal set of features that contributed to lung tumor categorization. This methodology is reported below.

#### Incremental Feature Selection

The predictor attributes generated by the Gain Ratio and CFS Subset Attribute Evaluator (Hybrid Feature Selection) method were later utilized for Incremental Feature Selection (IFS) [38]–[39] to determine the minimal and optimal set of features. On adding each feature, a new feature set was obtained and the k^th^ feature set could be stated as
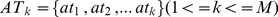
(6)


Where M denoted the total number of predictor subsets. On constructing each feature set, the predictor model was constructed and tested through Jack-knife cross-validation method. The MCC and Accuracy of cross-validation was measured, leading to the formation of the IFS table with the number of features and the classification accuracy they were able to generate. ‘AT_o_’ was the minimal and optimal feature set that achieved the highest MCC and accuracy.

In order to determine the best classification model for lung tumor classification [40], a total of five benchmark prediction techniques viz, Support Vector Machine [29], Random Forest [1], Nearest Neighbor algorithm [39], Bayesian Network Learning [22] and Random Committee (Ensemble classifier) [22] were analyzed and compared. Our results affirmed that Bayesian Network approach generated higher accuracy in tumor classification with the optimal feature set.

#### Bayesian Network Learning

The learning phase in this approach incorporated the process of finding an appropriate Bayesian network [41] given a data set D over R where R = {r_1_, r_n_}, n ≥1 was the set of input variables. The classification task consisted of classifying a variable V = v_0_ called the class variable (NSCLC/SCLC/COMMON) given a set of variables R = r_1_ . . . r_n_. A classifier C: r → v was a function that mapped an instance of ‘r’ to a value of ‘v’. The classifier was learned from a dataset D that consisted of samples over (r, v) [42]. A Bayesian network over a set of variables R was a network structure B_s_, a directed acyclic graph (DAG) over the set of variables R and a set of probability tables [43] was given by
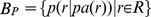
(7)


Where pa(r) was the set of parents of r in B_S_ and the network represented a probability distribution given by Eq. (8)
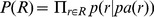
(8)


The inference made from the Bayesian Network [41]–[43] was to allocate the category with the maximum probability [44]. The Simple Estimator with the K2 local search method using Bayes Score were utilized (default parameters) for the execution of the algorithm in WEKA 3.7.7 [22]. The clustering methods are briefed about in the following section.

#### Supervised Clustering

Supervised clustering [45]–[47] deviated from unsupervised clustering in that it was applied on already categorized examples with the prime aim of detecting clusters that had high probability density with respect to a single class. Supervised clustering required the number of clusters to be kept to a minimum, and objects were assigned to clusters using the notion of closeness with respect to a given distance function [48]–[49]. Supervised clustering evaluated a clustering technique based on the following two criteria [47]–[49]:

- *Class impurity, Impurity(X):* It was measured by the percentage of marginal examples in the different clusters of a clustering X. A marginal example was an example that belonged to a class different from the most frequent class in its cluster.
- Number of clusters, k.

In this research we have compared the classes to cluster evaluation accuracy of seven clustering algorithms [22] namely Expectation-Maximization (EM) Algorithm, COBWEB [22], Hierarchical clustering, K-Means clustering, Farthest First Clustering, Density-Based clustering and Filtered Clustering. The number of clusters was automatically assigned in the COBWEB algorithm whereas the remaining algorithms allowed the user to select the desired number of clusters [22]. Some algorithms exhibited better performance on inclusion of all the attributes for clustering while the performance deteriorated on the hybrid feature selection datasets. The performance evaluation methods and parameters are briefed about in the subsequent sections.

#### Jack-knife Cross-Validation Test

Statistical prediction methods [50] were utilized for measuring the predictor performance in order to assess their efficiency in practical applications. In this study, the jack-knife cross validation method [50]–[51] was used for verification and validation of classifier accuracy since previous reports have stated it to be least arbitrary in nature and widely acclaimed by researchers and practitioners to estimate the performance of predictors. In jack-knife cross-validation [38]–[39] [52], each one of the statistical records in the training dataset was in turn singled out as a test sample and the predictor was trained by the remaining samples. During the jack-knifing process [23]–[24] [39], both the training dataset and testing dataset were actually open, and a statistical sample moved from one group to the other. In this research, the following indexes [50]–[52] were adopted to test the proposed methodology.
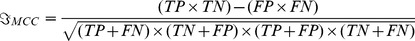
(9)

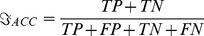
(10)where 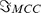
reflected the Mathews Correlation Coefficient; 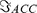
reflected the accuracy, i.e., the rate of correctly predicted lung cancer tumor class; TP, TN, FP and FN denoted the number of true positives, true negatives, false positives and false negatives, respectively.

## Experimental Results and Discussion

The experimental results are discussed in three sections. The foremost describes the ranking of the structural and physicochemical properties according to their gain ratio. The entire list of attributes was ranked and the file is provided as Table S1. The second section deals with the results of Incremental Feature Selection while the final section portrays the comparative performance of the benchmark classification models on the protein sequence properties in categorizing lung tumors.

### Hybrid Feature Selection

A total of 1497 attributes were initially loaded as the training data with 113 instances [17]–[18]. No records were duplicated and there were no missing values. On ranking the attributes by the Gain Ratio criterion, a total of 134 attributes were assigned a gain ratio greater than zero. The CFS subset evaluator returned 39 features as the most optimal subset that was highly correlated to the target class but least correlated to each other. These features were then utilized for the Incremental feature Selection process. The results of the Hybrid Feature Selection techniques are given as Table S1.

### Incremental Feature Selection

**Figure 2:**
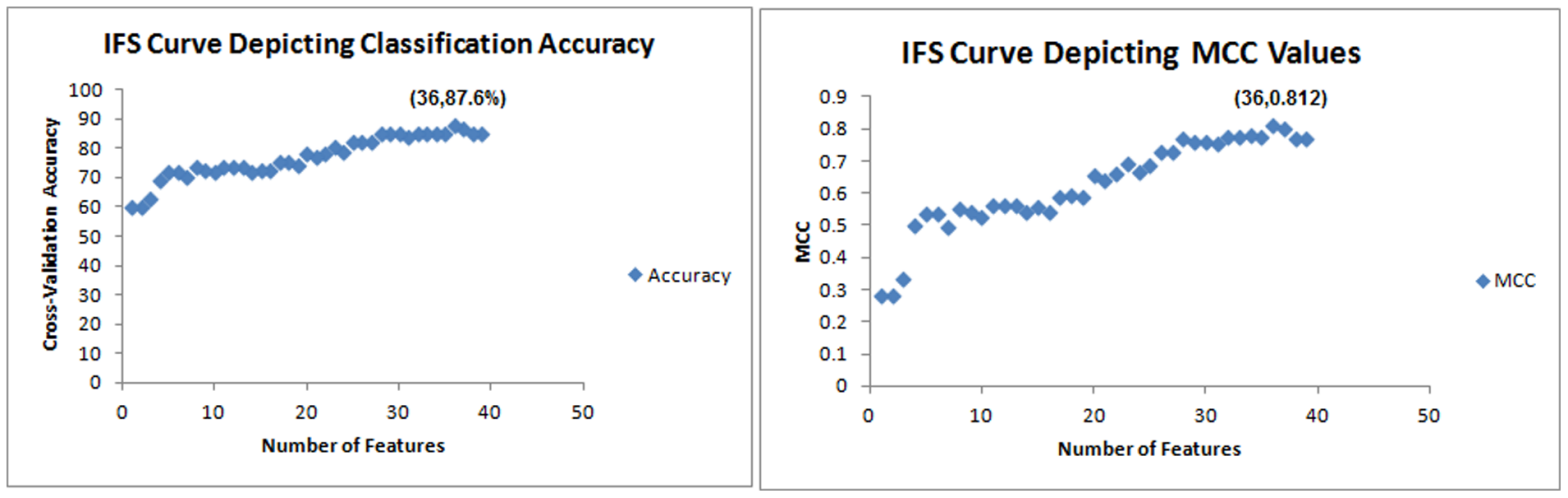
The IFS curves depicting classification accuracy and MCC in lung tumor categorization. (A) The IFS curve generated using Classification Accuracy in Lung Tumor categorization. The x-axis represented the number of features while the y-axis represented the jack-knife cross-validation accuracy. The peak of classification accuracy attained was 87.6% with 36 features. The top 36 features derived by Hybrid Feature Selection (Gain Ratio +CFS Subset) approach form the optimal feature set. (B) The IFS curve generated using MCC values obtained from classification algorithms. The peak of MCC is 0.812 with 36 features. The top 36 features derived by the Hybrid Feature Selection approach (Gain Ratio + CFS Subset) formed the optimal feature set.

The ranked attributes from the CFS subset evaluator were then input in the descending order of their rank to the classifier. At each attribute entry, the MCC and accuracy of the classifier on Jack-knife test was calculated. The Bayesian Network Learning was found to give the highest prediction MCC of 0.812 and accuracy of 87.6% with 36 features. The IFS curves generated on classifier accuracy and the corresponding MCC is represented in Figure 2. The optimal prediction accuracy with the proposed methodology for each feature subset is given in Table 1. The complete results of Incremental Feature Selection process on all the three Hybrid Feature Selection datasets are given in Table S2.

**Table 1:** Optimal classification accuracy with filtered subsets and IFS.

*Hybrid Feature Selection Technique*	*Features*	*Classification Algorithm*	*Jack-knife Cross-Validation Accuracy (%)*
Gain Ratio + CFS Subset	36		87.6
Information Gain +CFS Subset	32	Bayesian Network	85
Symmetric Uncertainty + CFS Subset	29		85.8

### Classifier Models

Benchmark classification models that have been reported [14] [38]–[39] [53]–[54] to generate high accuracy in classification of biological data were compared to determine the optimal prediction technique that generated highest accuracy in prediction. The comparative performance of the classification models with the feature set generated by the Hybrid Feature Selection technique is depicted in Table 2. The performance is compared based on the MCC and prediction accuracy.

**Table 2:** Comparison of predictor models in lung cancer tumor categorization.

*S.No*	*Hybrid Feature Selection Technique*	*Classifier*	*Training Phase*		*Prediction Phase*	
			*MCC*	*Accuracy*	*MCC*	*Accuracy*
1	Gain Ratio + CFS	Bayesian Network	0.895	92.9	**0.77**	**85**
2	Subset Evaluator	Random Forest	1	100	0.652	78.8
3		Nearest Neighbor	1	100	0.507	69
4		Support Vector Machine	0.856	91.2	0.603	76.1
5		Random Committee	1	100	0.484	69
1	Information Gain +	Bayesian Network	0.895	92.9	**0.77**	**85**
2	CFS SubsetEvaluator	Random Forest	1	100	0.61	76.1
3		Nearest Neighbor	1	100	0.52	69.9
4		Support Vector Machine	0.856	91.2	0.603	76.1
5		Random Committee	1	100	0.553	72.6
1	Symmetric	Bayesian Network	0.895	92.9	**0.77**	**85**
2	Uncertainty + CFS	Random Forest	1	100	0.521	71.7
3	Subset Evaluator	Nearest Neighbor	1	100	0.52	69.9
4		Support Vector Machine	0.84	90.3	0.603	76.1
5		Random Committee	1	100	0.62	77

### Clustering Models

This study utilized seven clustering algorithms [22] in order to compare their performance in categorizing the classes of lung tumors based on the attribute values. The results of generating the clustering algorithms on the dataset before and after performing hybrid feature selection are presented. The classes to cluster evaluation results are portrayed in Table 3. It is evident from the tabulated results that clustering algorithms were not useful in providing any new idea on the attribute significance in detecting clusters since their performance accuracy was substantially low. The discussions on the data and the results are presented in the ensuing section.

**Table 3:** Classes to cluster evaluation.

*S.No*	*Clustering Models*	*Classes to Cluster Evaluation Accuracy (%)*	
		*Pre- Hybrid feature selection*	*Post- Hybrid feature selection*
1	E-M Algorithm	52.2124	51.3274
2	COBWEB	2.6549	5.3097
3	K-Means	53.0973	51.3274
4	Hierarchical Clustering	51.3274	51.3274
5	Density Based Clustering	53.0973	52.2124
6	Filtered Clustering	53.0973	51.3274
7	Farthest First Clustering	48.6726	46.0176

## Discussion

### Influence of Structural and Physicochemical Properties

**Figure 3:**
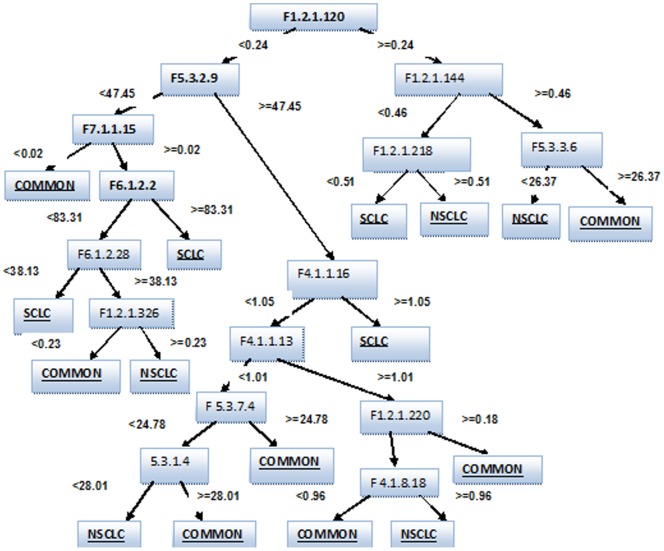
Decision tree model obtained by the Random Forest classifier.

There have been several researches on lung cancer classification [55]–[65] but the only previous computational study on the influence of protein sequence based structural and physicochemical properties in categorization of lung tumors was done by Hosseinzadeh et.al [1] who utilized the decision tree generated by the Random Forest classifier to identify the contributing attributes. In this study, we utilized the smallest tree among the 10 decision tree models generated by the Random Forest classifier [66] on the training dataset in order to identify the most contributing attributes to lung tumor classification. Albeit the Random Committee algorithm also depicted 100% accuracy and a high MCC of 1 in the training phase, the results obtained on Jack-knife cross-validation were not as high as the Random Forest Model. The decision tree model with the smallest number of nodes generated by the Random Forest on the training dataset is portrayed in Figure 3. The visualization of this tree made it easier to identify the composition of each protein property in the different types of lung cancer tumors, thus providing a source for drug design targeting the protein composition.

The following novel insights on the protein properties were gained from the Random Forest Model with a new set of discriminative features being reported for the first time in discriminating the lung tumor classes.

- Dipeptide composition was the most discriminating feature among the classes. F1.2 [Dipeptide Composition], F5.3 [Distribution Descriptor], F4.1 [Geary Auto-correlation] and F6.1 [Sequence order coupling number] were the subsequent significant protein properties used by the Random Forest Model to discriminate the lung tumor classes.
- A low value of the F5.3.2 [Normalized vdW volumes] and F [7.1] pseudo amino-acid composition moved the records into the COMMON class. A high F5.3.1 [distribution of hydrophobicity] and F5.3.3 [distribution of polarity] was found among the genes common in both classes of tumors whereas a lower concentration of the same was found among the NSCLC tumor genes. This directs molecular research to design drugs that would lower the distribution of hydrophobicity and polarity while raising the normalized vdW volumes and pseudo amino-acid composition to target the COMMON classes of tumors.
- A high dipeptide composition was characteristic of the NSCLC genes and a relatively low value represented the SCLC tumors. A high concentration of F5.3.1 [Distribution of hydrophobicity] and F5.3.7 [distribution of Solvent Accessibility] was evident in the COMMON classes of tumors. These findings suggest designing drugs that raise dipeptide composition to aid in cure of SCLC tumors and drugs that lower the dipeptide composition to cure NSCLC tumors. Moreover design of drugs that lower the distribution of hydrophobicity and solvent accessibility could aid in curing tumors of both kinds.

It was evident that a strict demarcation among the tumor categories was a complicated task since many properties were found to exhibit similar composition in both the tumor classes. However the proposed methodology was found to differentiate between the tumor classes with a high MCC of 0.812 and classification accuracy of 87.6%, the highest reported thus far in protein –property based lung tumor categorization.

### Comparison to Previous Work

As stated earlier, the only previous computational study on lung tumor categorization based on the protein sequence-based structural and physicochemical properties was reported by Hosseinzadeh et.al [1] that made a comparison of ten different feature selection techniques and reported the feature set generated by the Gain Ratio criterion to generate optimal 10-fold cross validation accuracy of 86% with the Random Forest classifier. Their methodology incorporated 114 sequences with 30 genes in the NSCLC class, 59 in the SCLC and 25 in the COMMON class of tumors. Moreover their methodology also involved extensive data cleaning and pre-processing. Here we made use of the 113 sequences [16]–[18] from the KEGG gene sets corresponding to the NSCLC and SCLC tumor classes and segregated the genes under the three classes viz, NSCLC, SCLC and COMMON. The number of records summed up to 113 with 29 genes [16]–[17] in the NSCLC class. This study was aimed at identifying the minimal and optimal set of features to categorize the lung tumor classes for use in diagnostic practice and drug design. Hence we used the Gain Ratio criterion, Information Gain criterion and Symmetric Uncertainty to rank the features and then applied the Correlation Feature Subset evaluator [22] with a search termination threshold of 5 and Best First Search approach to identify the smallest subset of features with a high correlation to the target class and least correlation to each other. This resulted in a feature subset with 39 features. On comparing the jack-knife cross-validation accuracy of five benchmark classification models, the Bayesian Network Learning algorithm was found to generate the highest MCC of 0.77 with an accuracy of 85% with all the three hybrid feature selection subsets. On applying Incremental Feature Selection we obtained the most optimal feature set of 36 features (feature subset of Gain Ratio + CFS) generating an accuracy of 87.6%.

The previous work by Hosseinzadeh et.al reported a high accuracy of 86% only on the cleaned data after removal of duplicate records, correlated records and based on the standard deviation values. When considering the same data, our proposed work has achieved a higher accuracy with the original, unmodified data thus saving computational time by the elimination of the data cleaning process. In order to bring out the comparison more clearly we have identified the accuracy of Random Forest with Gain Ratio (previously proposed classifier model) on the original data which was able to generate an optimal accuracy of only 79.6% with 26 features from the Gain Ratio –CFS feature set compared to our proposed method which produced 87.6% accuracy with 36 features from the same feature subset. We believe our proposed methodology can easily be extended to classify and discriminate between other oncogenic tumors since the original data was retained for computational analysis. However the previous method appears to have generated a high accuracy (86%) only on the cleaned data which makes it a limitation when extending the methodology to other cancer datasets. Moreover the previously proposed model would entail additional data pre-processing time when applied to new cancer datasets.

### Comparison with Other Methods

**Figure 4:**
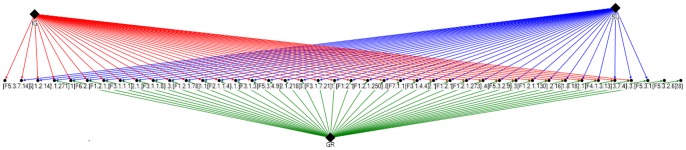
Feature relevance graph. The hybrid feature selection techniques are represented as solid diamonds. The optimal features filtered by each technique are represented by directed edges from the technique to the feature. Results of each hybrid feature selection technique are represented in different colors.

We compared three feature selection methods [22] namely Information Gain, Symmetric Uncertainty and Gain Ratio. We applied CFS Subset evaluator on all the feature sets ranked by the three algorithms. All the five benchmark classification algorithms [67]–[68] were applied on the reduced feature datasets. The results are tabulated in Table 2. All the three predictor methods displayed consistently high accuracy with the Bayesian Network prediction technique. The optimal accuracy was obtained only during the process of Incremental Feature Selection with the Gain Ratio and CFS subset evaluator combination which attained an improved accuracy of 87.6% with 36 features. Albeit the Bayesian Network learning algorithm showed consistent accuracy with the reduced feature sets of the Information Gain and Symmetric Uncertainty ranked features, yet during the process of Incremental Feature Selection, substantial decline in accuracy was apparent with the Information Gain and Symmetric Uncertainty subsets as detailed in the Table S2. Hence the Gain Ratio based ranking of features was considered to be the most optimal feature set for lung tumor categorization. The features selected by all the three hybrid feature selection techniques and the commonality among the selected features are displayed as a graph using NodeXL graph visualization software [69] in Figure 4. On careful analysis of the graphical representation of the feature subsets, it could be concluded that many features were commonly filtered by all the three hybrid feature selection techniques and hence reasonably similar performance accuracy was evident across the filtered subsets. However the process of Incremental Feature Selection disclosed the optimal and minimal feature set required for optimum prediction accuracy.

### Benefits of the Bayesian Network Learning Algorithm

Bayesian Networks have been used in several [70]–[73] clinical prediction problems. Previous research has stated that a Bayesian network is a mathematically rigorous way to model a domain problem, being flexible and adaptable to available knowledge, and computationally efficient [72] [74]–[75]. Some notable features of Bayesian Networks [44] for use in clinical prediction are narrated below.

- Bayes net only relates nodes that are probabilistically related by some sort of causal dependency. This eliminates the need to store all possible configurations of states. The algorithm stores and works with all possible combinations of states between sets of related parent and child nodes that greatly reduce computational complexity.
- Bayes Net utilizes expert knowledge and data to build models dynamically. It allows both backward and forward reasoning.

The medical domain is one research area where expert knowledge always has room for improvement and backward reasoning is a definite requirement. Hence application of computational techniques like Bayesian Networks in discriminating and classifying tumor classes based on protein sequence based physicochemical properties is expected to advance the current state of molecular and biological analysis of oncogenic tumor classes for drug design.

## Conclusion

Research on the utilization of computational techniques and predictions on clinical and biological data has intensified in the recent past owing to the fact that most wet-lab experiments consumed more human expertise, time and capital with irresolute rewards. This research was aimed at identifying the minimal and optimal set of protein sequence based structural and physicochemical properties in lung tumor categorization into NSCLC, SCLC and the COMMON tumor classes. The findings of this study are believed to be both a computational and biological advancement, the former revealing a new combination of feature selection and prediction techniques for categorizing tumor classes with enhanced accuracy and the latter acquiring information on protein properties prevalent in lung tumors that could aid in diagnostic practice and drug design. Possible extensions to this work would involve application of this novel computational framework in categorization of other oncogenic tumors and detecting properties that could be targeted for cancer therapy. Moreover computational advancement would require improving the prediction accuracy of the proposed methodology by possible updations to the existing algorithms.

## Supporting Information

File S1 **Attribute description file.**
(DOC)

File S2 **Pre-processed protein based structural and physicochemical data.**
(TXT)

Table S1 **Hybrid feature selection results.**
(XLS)

Table S2 **Incremental feature selection results.**
(XLS)

## References

[1] Hosseinzadeh F, Ebrahimi M, Goliaei B, Shamabadi N (2012) Classification of Lung Cancer Tumors Based on Structural and Physicochemical Properties of Proteins by Bioinformatics Models. PLoS ONE 7(7): e40017 doi:10.1371/journal.pone.0040017. 22829872 10.1371/journal.pone.0040017 PMC3400626

[2] American Cancer Society. Available: www.cancer.org/research/cancerfactsfigures/acspc-031941. Accessed: 2012 December 15.

[3] Lung Cancer Alliance website. Available: www.lungcanceralliance.org. Accessed: 2012 December 10.

[4] Li H, Sun L, Tang Z, Fu L, Xu Y, et al (2012) Overexpression of TRIM24 Correlates with Tumor Progression in Non-Small Cell Lung Cancer. PLoS ONE 7(5): e37657 doi:10.1371/journal.pone.0037657. 22666376 10.1371/journal.pone.0037657 PMC3364288

[5] Mehan MR, Ayers D, Thirstrup D, Xiong W, Ostroff RM, et al (2012) Protein Signature of Lung Cancer Tissues. PLoS ONE 7(4): e35157 doi:10.1371/journal.pone.0035157. 22509397 10.1371/journal.pone.0035157 PMC3324437

[6] West L, Vidwans SJ, Campbell NP, Shrager J, Simon GR, et al (2012) A Novel Classification of Lung Cancer into Molecular Subtypes. PLoS ONE 7(2): e31906 doi:10.1371/journal.pone.0031906. 22363766 10.1371/journal.pone.0031906 PMC3283716

[7] Hou J, Aerts J, Den Hamer B, Van IJcken W, Den Bakker M, et al (2010) Gene Expression-Based Classification of Non-Small Cell Lung Carcinomas and Survival Prediction. PLoS ONE 5(4): e10312 doi:10.1371/journal.pone.0010312. 20421987 10.1371/journal.pone.0010312 PMC2858668

[8] Lin Q, Peng Q, Yao F, Pan X-F, Xiong L-W, et al (2012) A Classification Method Based on Principal Components of SELDI Spectra to Diagnose of Lung Adenocarcinoma. PLoS ONE 7(3): e34457 doi:10.1371/journal.pone.0034457. 22461913 10.1371/journal.pone.0034457 PMC3312904

[9] Chopra P, Lee J, Kang J, Lee S (2010) Improving Cancer Classification Accuracy Using Gene Pairs. PLoS ONE 5(12): e14305 doi:10.1371/journal.pone.0014305. 21200431 10.1371/journal.pone.0014305 PMC3006158

[10] Dagliyan O, Uney-Yuksektepe F, Kavakli IH, Turkay M (2011) Optimization Based Tumor Classification from Microarray Gene Expression Data. PLoS ONE 6(2): e14579 doi:10.1371/journal.pone.0014579. 21326602 10.1371/journal.pone.0014579 PMC3033885

[11] Markey MK, Tourassi GD, Floyd CE Jr (2003) Decision tree classification of proteins identified by mass spectrometry of blood serum samples from people with and without lung cancer. Proteomics 3: 1678–1679. 12973724 10.1002/pmic.200300521

[12] Yamagata N, Shyr Y, Yanagisawa K, Edgerton M, Dang TP, et al (2003) A training-testing approach to the molecular classification of resected non-small cell lung cancer. Clin Cancer Res 9: 4695–4704. 14581339

[13] Marchevsky AM, Tsou JA, Laird-Offringa IA (2004) Classification of individual lung cancer cell lines based on DNA methylation markers: use of linear discriminant analysis and artificial neural networks. J Mol Diagn 6: 28–36. 14736824 10.1016/S1525-1578(10)60488-6 PMC1867460

[14] Guan P, Huang D, He M, Zhou B (2009) Lung cancer gene expression database analysis incorporating prior knowledge with support vector machine-based classification method. J Exp Clin Cancer Res 28: 103. 19615083 10.1186/1756-9966-28-103 PMC2719616

[15] Nguyen AN, Lawley MJ, Hansen DP, Bowman RV, Clarke BE, et al (2010) Symbolic rule-based classification of lung cancer stages from free-text pathology reports. J Am Med Inform Assoc 17: 440–445. 20595312 10.1136/jamia.2010.003707 PMC2995652

[16] Gene Set Enrichment Analysis Data: Gene Sets. Available: http://www.broadinstitute.org/cancer/software/gsea. Accessed 2012 Dec 12.

[17] KEGG (Kyoto Encyclopedia of Genes and Genomes). Available: http://www.kegg.jp/. Accessed 2012 Nov 30.

[18] Gene Card Database. Available: www.genecards.org. Accessed: 2012 Nov 25.

[19] Universal Protein Resource. Available: www.uniprot.org. Accessed:2012 Nov 27.

[20] Rao HB, Zh Fu, Yang GB, Li ZR, Chen YZ (2011) Update of PROFEAT: a web server for computing structural and physicochemical features of proteins and peptides from amino acid sequence. Nucleic Acids Res. Jul 1, 2011; 39(Web Server issue): W385–90. 10.1093/nar/gkr284 PMC3125735 21609959

[21] ZR Li, HH Lin, LY Han, L Jiang, X Chen, YZ Chen (2006) PROFEAT: A Web Server for Computing Structural and Physicochemical Features of Proteins and Peptides from Amino Acid Sequence. Nucleic Acids Res. Jul 1, 2006; 34(Web Server issue): W32–7. 10.1093/nar/gkl305 PMC1538821 16845018

[22] Waikato Environment for Knowledge Analysis (WEKA) Machine Learning Tool, Available: http. Accessed 2012 Dec 1.

[23] Huang T, Shi XH, Wang P, He Z, Feng KY, et al (2010) Analysis and prediction of the metabolic stability of proteins based on their sequential features, sub cellular locations and interaction networks. PLoS ONE 2010 5(6): e10972. 10.1371/journal.pone.0010972 PMC2881046 20532046

[24] Huang T, Wang P, Ye ZQ, Xu H, He Z, et al (2010) Prediction of Deleterious Non-Synonymous SNPs Based on Protein Interaction Network and Hybrid Properties. PLoS ONE 5(7): e11900. 20689580 10.1371/journal.pone.0011900 PMC2912763

[25] Baldi P, Brunak S, Chauvin Y, Andersen CA, Nielsen H (2000) Assessing the accuracy of prediction algorithms for classification: An overview. Bioinformatics 16: 412–424. 10871264 10.1093/bioinformatics/16.5.412

[26] Jacob SG, R Geetha Ramani (2011) Discovery of Knowledge Patterns in Clinical Data through Data Mining Algorithms: Multi-class Categorization of Breast Tissue Data. International Journal of Computer Applications (IJCA) 32(7): 46–53 DOI:10.5120/3920-5521. Published by Foundation of Computer Science, New York, USA.

[27] Jacob SG, Ramani RG, Nancy P (2011) Feature Selection and Classification in Breast Cancer Datasets through Data Mining Algorithms. Proceedings of the IEEE International Conference on Computational Intelligence and Computing Research (ICCIC'2011), Kanyakumari, India, IEEE Catalog Number: CFP1120J-PRT, 978-1-61284-766-5: 661–667.

[28] Selvakuberan K, Indradevi M, Rajaram R (2008) Combined Feature Selection and classification – A novel approach for the categorization of web pages. Journal of Information and Computing Science Vol. 3, No. 2, 2008, 083–089.

[29] Jacob SG, Ramani RG, Nancy P (2012) Efficient Classifier for Classification of Hepatitis C Virus Clinical Data through Data Mining Algorithms and Techniques. Proceedings of the International Conference on Computer Applications, Pondicherry, India, Techno Forum Group, India. ISBN: 978-81-920575-8-3: DOI: 10.73445/ISBN_0768, ACM#.dber.imera.10.73445.

[30] Jacob SG, Ramani RG(2012) Mining of Classification Patterns in Clinical Data through Data Mining Algorithms. Proceedings of the International Conference on Advances in Computing, Communications and Informatics. Pages 997-1003 ACM New York, NY, USA ©2012 ISBN: 978-1-4503-1196-0 doi>10.1145/2345396.2345557.

[31] Jacob SG, Ramani RG (2012) Evolving Efficient Classification Rules from Cardiotocography Data through Data Mining Methods and Techniques. European Journal of Scientific Research, Print ISSN: 1450-202X, E-ISSN 1450-216X Vol.78 No.3 468–480.

[32] Cios K, Pedrycz W, Swiniarski R (1998) Data Mining Methods for Knowledge Discovery. Boston: Kluwer Academic Publishers.

[33] Mitchell T (1997) Machine Learning, Tata Mc-Graw Hill. 414 pages. ISBN 0070428077.

[34] Han J, Kamber M (2000) Data Mining: Concepts and Techniques. Morgan Kaufmann Publishers.

[35] Earl Harris Jr (2003) Information Gain Versus Gain Ratio: A Study of Split-Method Biases. 2001 The MITRE Corporation. All Rights Reserved.

[36] Hall M(1999) Correlation-based Feature Selection for Machine Learning, PhD Thesis.

[37] Manning CD, Raghava P, Schutze H (2008) Introduction to Information Retrieval. Cambridge University Press. ISBN 978-0-521-86571-5.

[38] Huang T, Wan S, Xu Z, Zheng Y, Feng KY, et al (2011) Analysis and prediction of translation rate based on sequence and functional features of the mRNA. PLoS ONE 2011 6(1): e16036. 10.1371/journal.pone.0016036 PMC3017080 21253596

[39] Huang T, Niu S, Xu Z, Huang Y, Kong X, et al (2011) Predicting the Transcriptional Activity of Multiple Site p53 mutants based on Hybrid Properties. 6(8): e22940 doi:10.1371/journal.pone.0022940. 10.1371/journal.pone.0022940 PMC3152557 21857971

[40] Crimins F (2003) Higher Dimensional Approach for Classification of Lung Cancer Microarray Data. CAMDA 03.

[41] Heckerman D (1995) A Tutorial on Learning with Bayesian Networks, Technical Report, March, 1995, Microsoft.

[42] Pourret O, Naim P, Marcot B (2008) Bayesian Networks: A Practical Guide to Applications. Chichester, UK: Wiley. ISBN 978-0-470-06030-8.

[43] Friedman N, Linial M, Nachman I, Pe'er D (August 2000) Using Bayesian Networks to Analyze Expression Data. Journal of Computational Biology (Larchmont, New York: Mary Ann Liebert, Inc.) 7 (3/4): 601–620 doi:10.1089/106652700750050961. ISSN 1066-5277.PMID 11108481 10.1089/106652700750050961 11108481

[44] Kotsiantis SB (2007) Supervised Machine Learning: A Review of Classification Techniques. Informatica 31249–268.

[45] Marina M (2003) Comparing Clustering by the Variation of Information. Learning Theory and Kernel Machines: 173–187.

[46] Kraskov A, Stögbauer H, Andrzejak RG, Grassberger P (2003) Hierarchical Clustering Based on Mutual Information. ArXiv q-bio/0311039.

[47] Eick CF, Zeidat N, Zhao Z (2004) Supervised Clustering – Algorithms and Benefits. Proceedings of the 16th IEEE International Conference on Tools with Artificial Intelligence (ICTAI'04) Boca Raton, Florida, November 2004 774–776.

[48] Rand WM (1971) Objective criteria for the evaluation of clustering methods. Journal of the American Statistical Association (American Statistical Association) 66 (336): 846–850 Doi:10.2307/2284239. JSTOR 2284239.

[49] Guyon I, von Luxburg U, Williamson RC (2009) Clustering: Science or Art? In NIPS Workshop on Clustering Theory.

[50] Kohavi R (1995) A study of cross-validation and bootstrap for accuracy estimation and model selection. Proceedings of the Fourteenth International Joint Conference on Artificial Intelligence 2 (12): 1137–1143.

[51] Picard R, Cook D (1984) Cross-Validation of Regression Models. Journal of the American Statistical Association 79 (387): 575–583.

[52] Deng H, Runger G, Tuv E (2011) Bias of importance measures for multi-valued attributes and solutions. Proceedings of the 21st International Conference on Artificial Neural Networks (ICANN2011): 293–300.

[53] Zhou XB, Chen C, Li ZC, Zou XY (2007) Using Chou's amphiphilic pseudo amino acid composition and support vector machine for prediction of enzyme subfamily classes. Journal of Theoretical Biology 248: 546–551. 17628605 10.1016/j.jtbi.2007.06.001

[54] Iba W, Langley P (1992) Induction of One-Level Decision Trees, in ML92: Proceedings of the Ninth International Conference on Machine Learning, Aberdeen, Scotland, 1–3 July 1992, San Francisco, CA: Morgan Kaufmann, 233–240.

[55] Ebrahimi M, Ebrahimie E, Shamabadi N (2010) Are there any differences between features of proteins expressed in malignant and benign breast cancers? J Res Med Sci 15: 299–309. 21526102 PMC3082830

[56] Furney SJ, Higgins DG, Ouzounis CA, Lopez-Bigas N (2006) Structural and functional properties of genes involved in human cancer. BMC Genomics 7: 3. 16405732 10.1186/1471-2164-7-3 PMC1373651

[57] Aragues R, Sander C, Oliva B (2008) Predicting cancer involvement of genes from heterogeneous data. BMC Bioinformatics 9: 172. 18371197 10.1186/1471-2105-9-172 PMC2330045

[58] Travis WD (2011) Classification of lung cancer. Semin Roentgenol 46: 178–186. 21726702 10.1053/j.ro.2011.02.003

[59] Nevins JR (2011) Pathway-based classification of lung cancer: a strategy to guide therapeutic selection. Proc Am Thorac Soc 8: 180–182. 21543798 10.1513/pats.201006-040MS PMC3131836

[60] Raj V, Bajaj A, Entwisle JJ (2011) Implications of new (seventh) TNM classification of lung cancer on general radiologists–a pictorial review. Curr Probl Diagn Radiol 40: 85–93. 21266272 10.1067/j.cpradiol.2010.02.002

[61] Wrona A, Jassem J (2010) The new TNM classification in lung cancer. Pneumonol Alergol Pol 78: 407–417. 21077033

[62] Kligerman S, Abbott G (2010) A radiologic review of the new TNM classification for lung cancer. AJR Am J Roentgenol 194: 562–573. 20173129 10.2214/AJR.09.3354

[63] Nie GJ, Feng FF, Wu YJ, Wu YM (2009) Diagnosis and prediction of lung cancer through different classification techniques with tumor markers. Zhonghua Lao Dong Wei Sheng Zhi Ye Bing Za Zhi 27: 257–261. 19538834

[64] Yang Y, Pan QJ, Teng MF, Li ZL, Zhao LL, et al (2008) Application of protein markers in combination with ThinPrep bronchial brush cytology in classification of lung cancer subtypes. Zhonghua Zhong Liu Za Zhi 30: 616–619. 19102942

[65] Barash O, Peled N, Tisch U, Bunn PA Jr, Hirsch FR, et al. (2011) Classification of lung cancer histology by gold nanoparticle sensors. Nanomedicine: Nanotechnology, Biology, and Medicine 8 (2012) 580–589. 10.1016/j.nano.2011.10.001 PMC4745892 22033081

[66] Leo Breiman, Adele Cuttler, Random Trees. Available: http://www.stat.berkeley.edu/users/breiman/RandomForests/. Accessed 2012 Dec 10.

[67] Jacob SG, Ramani RG (2013) Design and Implementation of a Clinical Data Classifier: A Supervised Learning Approach. Res J Biotech. Vol. 8(2): 16–26.

[68] Geetha Ramani R, Jacob SG (2013) Prediction of P53 Mutants (Multiple Sites) Transcriptional Activity Based on Structural (2D & 3D) Properties. PLoS ONE 8(2) : e55401. doi:10.1371/journal.pone.0055401. 10.1371/journal.pone.0055401 PMC3572112 23468845

[69] NodeXl Visualization Tool. Available: http://nodexl.codeplex.com/releases/view/96383. Accessed: 2012 Dec 12.

[70] Peter L (2004) Bayesian Analysis, Pattern Analysis and Data Mining in Health Care. Current Opinion in Critical Care 10: 399. 15385759 10.1097/01.ccx.0000141546.74590.d6

[71] Medical Inference by Network Integration of Temporal Data Using Bayesian Analysis. Available:http://www.minituba.org/docs/tutorial.php. Accessed 2012 Dec 10. 10.1093/bioinformatics/btm372 17644819

[72] Watt EW, Bui AAT (2008) Evaluation of a Dynamic Bayesian Belief Network to Predict Osteoarthritic Knee Pain Using Data from the Osteoarthritis Initiative, AMIA Annu Symp Proc. 2008 2008: 788–792. PMC2656041 18999030

[73] Li J, Serpen G, Selman S, Franchetti M, Riesen M, Schneider C (2010) Bayes Net Classifiers for Prediction of Renal Graft Status and Survival Period World Academy of Science, Engineering and Technology 39 144–150.

[74] Uebersax (2004). Genetic Counseling and Cancer Risk Modeling: An Application of Bayes Nets. Marbella, Spain: Ravenpack International.

[75] Jiang X, Cooper GF (July–August 2010) A Bayesian spatio-temporal method for disease outbreak detection. J Am Med Inform Assoc 17 (4): 462–71. 10.1136/jamia.2009.000356 PMC2995651 20595315

