# Supplementary material for: Improved Classification of Lung Cancer Tumors Based on Structural and Physicochemical Properties of Proteins Using Data Mining Models
Source: PLoS One. 2013 Mar 7;8(3):e58772. doi: 10.1371/journal.pone.0058772 (PMC3591381; doi:10.1371/journal.pone.0058772)
Supplement: File S1 — Attribute description file. (DOC) [file pone.0058772.s001.doc]

Structural and Physicochemical Protein Attribute Description Obtained from PROFEAT Web Server

| [F1] Aminoacid, dipeptide composition (%) |
| --- |
| [F1.1.1] Aminoacid composition (%) |
| [F1.1] Aminoacid composition (%) |
| [F1.2.1] Dipeptide composition (%) |
| [F1.2] Dipeptide composition (%) |
| [F2] Autocorrelation1 |
| [F2.1] Normalized Moreau-Broto autocorrelation |
| [F2.1.1] M-B autocorrelation Hydrophobicity Scale |
| [F2.1.2] M-B autocorrelation Flexibility Indices |
| [F2.1.3] M-B autocorrelation Polarizability |
| [F2.1.4 M-B autocorrelation Free Energy in water |
| [F2.1.5] M-B autocorrelation Residue Accessible Surface Area in Tripeptide |
| [F2.1.6] M-B autocorrelation Residue Volume |
| [F2.1.7] M-B autocorrelation Steric Parameter |
| [F2.1.8] M-B autocorrelation Relative Mutability |
| [F3] Autocorrelation2 |
| [F3.1] Moran autocorrelation |
| [F3.1.1] Moran autocorrelation Hydrophobicity Scale |
| [F3.1.2] Moran autocorrelation Flexibility Indices |
| [F3.1.3] Moran autocorrelation Polarizability |
| [F3.1.4] Moran autocorrelation Free Energy in water |
| [F3.1.5] Moran autocorrelation Residue Accessible Surface Area in Tripeptide |
| [F3.1.6] Moran autocorrelation Residue Volume |
| [F3.1.7] Moran autocorrelation Steric Parameter |
| [F3.1.8] Moran autocorrelation Relative Mutability |
| [F4] Autocorrelation3 |
| [F4.1] Geary autocorrelation |
| [F4.1.1] Geary autocorrelation Hydrophobicity Scale |
| [F4.1.2] Geary autocorrelation Flexibility Indices |
| [F4.1.3] Geary autocorrelation Polarizability |
| [F4.1.4] Geary autocorrelation Free Energy in water |
| [F4.1.5] Geary autocorrelation Residue Accessible Surface Area in Tripeptide |
| [F4.1.6] Geary autocorrelation Residue Volume |
| [F4.1.7] Geary autocorrelation Steric Parameter |
| [F4.1.8] Geary autocorrelation Relative Mutability |
| [F5] Composition, transition, distribution |
| [F5.1] Composition |
| [F5.1.1] Composition of Hydrophobicity |
| [F5.1.2] Composition of Normalized vdW volumes |
| [F5.1.3] Composition of Polarity |
| [F5.1.4] Composition of Polarizability |
| [F5.1.5] Composition of Charge |
| [F5.1.6] Composition of Secondary structure |
| [F5.1.7] Composition of Solvent accessibility |
| [F5.2] Transition |
| [F5.2.1] Transition of Hydrophobicity |
| [F5.2.2] Transition of Normalized vdW volumes |
| [F5.2.3] Transition of Polarity |
| [F5.2.4] Transition of Polarizability |
| [F5.2.5] Transition of Charge |
| [F5.2.6] Transition of Secondary structure |
| [F5.2.7] Transition of Solvent accessibility |
| [F5.3] Distribution |
| [F5.3.1] Distribution of Hydrophobicity |
| [F5.3.2] Distribution of Normalized vdW volumes |
| [F5.3.3] Distribution of Polarity |
| [F5.3.4] Distribution of Polarizability |
| [F5.3.5] Distribution of Charge |
| [F5.3.6] Distribution of Secondary structure |
| [F5.3.7] Distribution of Solvent accessibility |
| [F6] Sequenceorder |
| [F6.1] Sequence-order-coupling number |
| [F6.1.1] sequence-order-coupling numbers based on Schneider-Wrede distance |
| [F6.1.2] sequence-order-coupling numbers based on normalized Grantham chemical distance |
| [F6.2] Quasi-sequence-order descriptors |
| [F6.2.1] Quasi-sequence-order descriptors based on Schneider-Wrede distance |
| [F6.2.2] Quasi-sequence-order descriptors based on normalized Grantham chemical distance |
| [F7] Pseudo aminoacid composition |
| [F7.1] Pseudo aminoacid composition |
| [F7.1.1] Pseudoaminoacid composition descriptors based on The aminoacid composition and sequence order components |
